# Aqueous dispersions of few-layer-thick chemically modified magnesium diboride nanosheets by ultrasonication assisted exfoliation

**DOI:** 10.1038/srep10522

**Published:** 2015-06-04

**Authors:** Saroj Kumar Das, Amita Bedar, Aadithya Kannan, Kabeer Jasuja

**Affiliations:** 1 Discipline of Chemical Engineering, Indian Institute of Technology Gandhinagar, Ahmedabad, Gujarat 382424, India; 2 Department of Chemical Engineering, Indian Institute of Technology Kharagpur, Kharagpur, 721302, India

## Abstract

The discovery of graphene has led to a rising interest in seeking quasi two-dimensional allotropes of several elements and inorganic compounds. Boron, carbon’s neighbour in the periodic table, presents a curious case in its ability to be structured as graphene. Although it cannot independently constitute a honeycomb planar structure, it forms a graphenic arrangement in association with electron-donor elements. This is exemplified in magnesium diboride (MgB_2_): an inorganic layered compound comprising boron honeycomb planes alternated by Mg atoms. Till date, MgB_2_ has been primarily researched for its superconducting properties; it hasn’t been explored for the possibility of its exfoliation. Here we show that ultrasonication of MgB_2_ in water results in its exfoliation to yield few-layer-thick Mg-deficient hydroxyl-functionalized nanosheets. The hydroxyl groups enable an electrostatically stabilized aqueous dispersion and create a heterogeneity leading to an excitation wavelength dependent photoluminescence. These chemically modified MgB_2_ nanosheets exhibit an extremely small absorption coefficient of 2.9 ml mg^−1^ cm^−1^ compared to graphene and its analogs. This ability to exfoliate MgB_2_ to yield nanosheets with a chemically modified lattice and properties distinct from the parent material presents a fundamentally new perspective to the science of MgB_2_ and forms a first foundational step towards exfoliating metal borides.

Last decade has witnessed extensive research in the science of graphene and its analogous nanomaterials. Several families of layered materials, like transition metal dichalcogenides, metal halides, metal oxides, and layered double hydroxides have been exfoliated to yield nanostructures analogous to graphene^1^,^2^,^3^,^4^. Owing to their high aspect ratio and quasi two-dimensional (2-D) electron confinement, graphene and its analogs have proved to be prolific platforms to probe and utilize the fascinating science of carbon and other elements at the atomic level^1^,^4^,^5^,^6^,^7^.

Boron, carbon’s neighbour in the periodic table, has always sought immense interest from the scientific community on account of the rich properties it offers: low density, high melting point, ability to capture neutrons, and high chemical stability^8^,^9^,^10^,^11^. Several 1-D nanostructures constituted from boron and boron-based compounds have presented avenues for leveraging these properties^11^,^12^. However, the research on quasi 2-D boron-based nanostructures has been primarily limited to hexagonal boron nitride (*h*-BN) nanosheets synthesized by exfoliation of layered *h*-BN or chemical vapour deposition^2^,^7^,^12^,^13^,^14^,^15^,^16^. The recent report on synthesizing a 36-atom quasi-planar cluster of boron (with a hexagonal vacancy) as a potential basis for borophene (a planar sheet constituted from boron) showcases the rising interest in developing boron-based quasi 2-D nanostructures^11^,^17^. Demonstrating the viability of exfoliating a layered boron-based compound alternative to *h*-BN will significantly add to the current state of knowledge on quasi 2-D boron-based nanostructures.

In this pursuit of exploring layered boron-based compounds alternative to *h*-BN, we investigated the possibility of exfoliating magnesium diboride (MgB_2_): a layered compound with Mg atoms sandwiched in between 2-D boron honeycomb planes, the adjacent layers being held together by ionic bonding^18^,^19^,^20^,^21^ (Fig. 1a). Pristine MgB_2_ exhibits a range of extraordinary physico-chemical properties that are characteristic to the family of metal borides. The presence of metal-boron and boron-boron bonds renders high chemical stabilities, high melting points, ultra-mechanical hardness, and high electrical and thermal conductivities to the metal borides. It is expected that nano scaling the metal borides would enable utilizing these properties to their full potential^18^,^22^,^23^,^24^,^25^,^26^. Moreover, the structural resemblance of MgB_2_ to intercalated graphite and the presence of boron honeycomb planes make it an extremely promising candidate for potentially yielding nanostructures which can facilitate access to the quasi-planar form of boron.

We show that ultrasonication of MgB_2_ in the presence of water can result in its exfoliation to yield an aqueous dispersion of few-layer-thick nanosheets with micron scale lateral dimensions. These nanosheets are found to exhibit a magnesium deficient stoichiometry while being functionalized with hydroxyl groups. A net negative surface charge on these nanosheets facilitates electrostatic stabilization and formation of a stable aqueous dispersion (Fig. 1b). These water-dispersed nanosheets are found to be transparent in the visible region and exhibit a broad absorption between 190–400 nm with an extremely small absorptivity of 2.9 ml mg^−1^ cm^−1^. The functional groups are also shown to impart an excitation wavelength dependent photoluminescence to the nanosheets. This discovery provides a new perspective on the science of MgB_2_ that has been largely known for its superconductive properties. These results also set a platform for exploring the exfoliation of other layered metal borides.

## Exfoliation of MgB_2_

**Figure 1:**
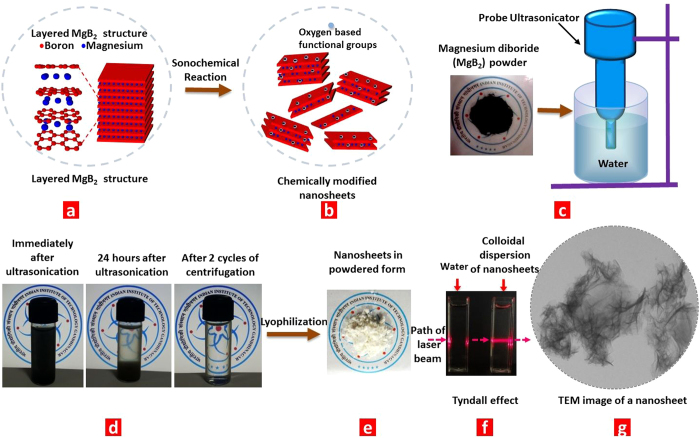
Liquid exfoliation route to synthesize chemically modified magnesium diboride nanosheets (CMMBs): **a,b**, Scheme representing the exfoliation of layered MgB_2_ to few-layer-thick chemically modified MgB_2_ nanosheets. **c**, Pristine MgB_2_ powder is exposed to ultrasonication using a probe ultrasonicator. **d**, Immediately after ultrasonication, the suspension exhibits a dark black color. As it is allowed to stand for 24 hours, the heavier particles sediment. These heavier particles are removed by mild centrifugation (2 cycles of 1500 rpm for 45 min each) to obtain a transparent homogeneous dispersion that is shown to contain nanosheets. **e**, The dispersion is lyophilized to obtain the nanosheets in a powdered form. **f**, Passing a laser beam results in a discernible track in the dispersion (Tyndall effect) confirming the colloidal nature of the dispersion. **g**, TEM image of a nanosheet from the dispersion.

The process of exfoliation was carried out by suspending 450 mg of MgB_2_ powder (–100 mesh size) in 150 ml of water and exposing it to ultrasonication for 30 minutes (probe ultrasonicator, amplitude: 30%, 10 sec on/off pulse) (Fig. 1c). This resulted in a dark black suspension, the black color being attributed to the color of pristine MgB_2_ powder. On allowing the suspension to stand for 24 hours, we observed sedimentation of dark pellets suggesting that the suspension consists of macroscopic aggregates. We pelleted out all such aggregates by mild centrifugation (2 cycles of 1500 rpm for 45 min, top half of centrifuged dispersion was collected after each cycle) to obtain 45 ml of homogeneous phase that appeared completely transparent to the naked eye (Fig. 1d). Irradiating a laser beam through this homogeneous phase left a discernible track suggesting the presence of a dispersed phase (Tyndall effect experiment, Fig. 1f). Upon lyophilisation, this dispersion resulted in a white powder (Fig. 1e), that was weighed to be ~60 mg suggesting the yield to be ~13%. This visible change in the color (from black to white) suggests chemical functionalization. As explained ahead, the dispersed phase is found to consist of few-layer-thick MgB_2_ nanosheets chemically modified with hydroxyl functional groups.

## Characterization

**Figure 2:**
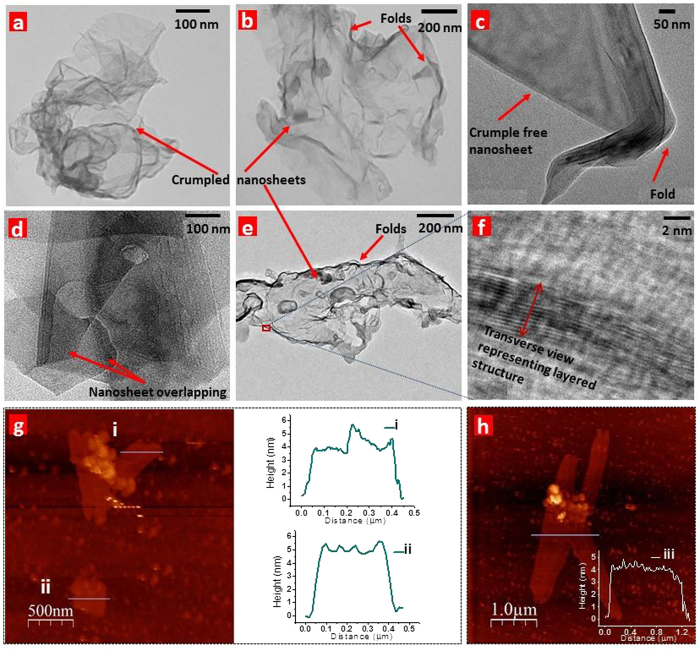
Transmission electron microscopy (TEM) and atomic force microscopy (AFM) images of the CMMB nanosheets. The nanosheets were immobilized from the aqueous disperion on copper grids supported with carbon films for electron microscopic visualization. **a**, Extremely crumpled nanosheet, **b**, Moderately crumpled nanosheet exhibiting folds, **c**, Crumple free nanosheet exhibiting a fold. **d**, HRTEM images of nanosheets overlapping each other. **e,** HRTEM image of a crumpled nanosheet. **f**, Transverse view of the selected edge of a nanosheet displaying intermittent layers. **g**, **h**, Tapping mode AFM images of the nanosheets deposited on mica substrate. The corresponding height profiles (shown in insets i, ii, and iii) indicate the thickness of nanosheets to be ~4–6 nm. These observations suggest the presence of few-layer-thick nanosheets.

We analysed the colloidal dispersion using transmission electron microscopy (TEM) and observed the presence of sheet-like nanostructures that resemble exfoliated graphite (Fig. 1g and Fig. 2, see Supplementary Information, Fig. S1 for more representative images). Some nanosheets appear extremely crumpled, some moderately crumpled, and some free of any crumples (Fig. 2a-c, see Supplementary Information, Fig. S2). Some nanosheets exhibit folds near their edges (Fig. 2b and c). The crumples and folds in the nanosheets could be an outcome of one or several of the following reasons: (i) immobilization from the aqueous solution on to the TEM support that involves uncontrolled capillary compression during rapid evaporation of water^27^,^28^; (ii) gaining thermodynamic stability (similar to the observation in graphene oxide that undergoes intrinsic bending and corrugation to stabilize its structure^28^,^29^); (iii) presence of oxy-functional groups (that is validated ahead). Similar crumples/wrinkles have been shown to arise upon the oxygen functionalization of graphene^30^. Schniepp *et al.*^28^,^31^ have explained that the oxy-functional groups create a strain on the nanosheet backbone, and in order to release this strain, a kink on the surface is created which makes the structure more favoured towards a wrinkled topology. Several other important factors that contribute to crumpling in nanosheets have been comprehensively summarized by Cheng *et al.* in reference to graphene oxide sheets^28^.

The high-resolution TEM (HRTEM) image in fig. 2f presents the transverse view of a nanosheet. The presence of alternating dark lines indicate the existence of a layered structure. Similar transverse views of nanosheets are included in Fig. S1 in Supplementary Information. The thickness of nanosheets was obtained through atomic force microscopy (AFM). Samples for AFM analysis were prepared by depositing the colloidal dispersion on a freshly cleaved mica substrate (see Methods). Figure 2g-h represent tapping mode AFM scans of the nanostructures. The corresponding height profiles are shown in insets i, ii, and iii; these indicate the nanosheets’ thickness to be ~4–6 nm suggesting that the nanosheets are few-layer-thick. Height profiles from other regions of the AFM scans are included in Supplementary Information, Fig. S2.

**Figure 3:**
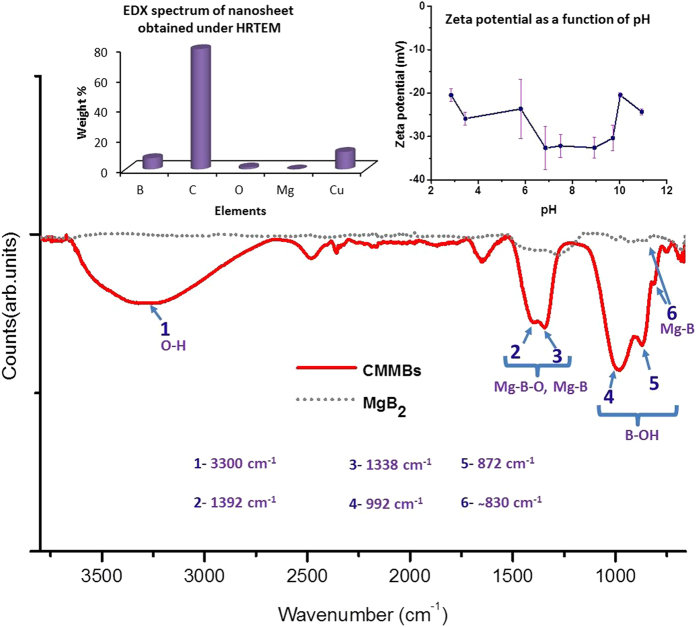
Chemical characterization of the nanosheets. ATR-FTIR spectrum obtained on a powdered sample of nanosheets (prepared by lyophilization). Band 1 at ~3300 cm^−1^ indicates the presence of hydroxyl functional groups suggesting a chemical modification of the parent layered structure. Top left inset shows the EDX analysis of nanosheets immobilized on a TEM grid. Top right inset shows the distribution of zeta potential of the colloidal dispersion over a range of pH values. The negative value of zeta potential indicates a net negative charge on the nanosheets while dispersed in an aqueous phase.

In order to develop insights on the chemical make-up of nanosheets, energy dispersive X-ray (EDX) spectra were recorded under HRTEM. The EDX spectrum (Fig. 3, top left inset) showed strong signals of carbon and copper (that are originating from the TEM grid); moderate signal of boron; and relatively weak signals of oxygen and magnesium (see Supplementary Information, Fig. S3 for similar EDX spectra). This observation indicates the presence of oxygen, and hence the possibility of nanosheets being functionalized. In order to understand the nature of functional groups, ATR-FTIR spectra were acquired on the powdered form of nanosheets (obtained after lyophilization) and pristine MgB_2_ powder (represented by solid and dotted lines respectively in Fig. 3). The FTIR spectrum of nanosheets exhibits a broad band at ~3300 cm^−1^ that indicates the presence of O-H functional groups^32^,^33^. The overlapping bands located at ~1392 cm^−1^ and ~1338 cm^−1^ are assigned to Mg-B and Mg-B-O bonds^32^,^34^. The weak band at ~830 cm^−1^ corresponds to the Mg-B bond^32^,^33^,^34^. The bands located at ~992 cm^−1^ and ~872 cm^−1^ are assigned to the in-plane bending and the out-of-plane bending of B-O-H bond respectively^33^. These two aforementioned peaks can also be attributed to the symmetric stretching (trigonal or tetrahedral mode) or the asymmetric stretching (tetrahedral mode) of the B-O-H bond^35^,^36^. The B-B in-plane stretching mode is reported to absorb at ~648 cm^−1^ 37, and thus couldn’t be detected in the spectrum range of 680 cm^−1^–3800 cm^−1^ that we recorded. The presence of B-B bonds was validated by acquiring Raman spectra and confirming the presence of E_2g_ mode (see Supplementary Information, Fig. S4c)^38^,^39^. These observations suggest that MgB_2_ lattice has been chemically modified with hydroxyl functional groups.

In order to understand the origin of this chemical modification, we referred to the valency charge density maps of MgB_2_. These maps indicate that the energetic cost to break various bonds in MgB_2_ is as follows: 3.43 eV/atom (B-B), 1.16 eV/atom (Mg-B), 0.45 eV/atom (Mg-Mg)^19^,^20^. This suggests that Mg atoms are energetically more favourable to be displaced in comparison to boron^19^,^20^. During ultrasonication, the liquid cavitation induces stress waves and micro jets which will generate normal and shear forces on the layered MgB_2_ crystals^40^. Under the effect of these forces, some Mg atoms are expected to be displaced. This displacement of Mg atoms is supported by the ICP-AES analysis on the nanosheets that is used to determine the stoichiometric ratios of Mg:B. A much lesser stoichiometric ratio of Mg:B is exhibited by the nanosheets as compared to pristine MgB_2_ (as shown in Table 1, details of the calculations are shown in Supplementary Information, S4). Before this analysis, the colloidal dispersion was dialysed to ensure that the ICP-AES signals are solely arising from the nanosheets and not any residual ions. The loss of Mg is also evidenced by the low Mg content in the EDX spectrum of nanosheets as compared to the Mg content in the EDX spectrum of pristine MgB_2_ (Supplementary Information, Fig. S4 a and b).

These observations suggest that during ultrasonication, a fraction of Mg atoms are being extracted from the interspaces of boron honeycomb planes. This is likely to cause a loss of interlayer binding and delamination of MgB_2_. During TEM imaging, we came across rare instances that capture this delamination (see Supplementary Information, Fig. S5). The loss of valence Mg atoms is likely being compensated by a subsequent gain of hydroxyl ligands. These ligands could be contributed by the water molecules similar to the acquisition of hydroxyl groups by the exfoliated Ti_3_C_2_ layers formed upon extraction of Al from Ti_3_AlC_2_^41^. The feasibility of B to acquire hydroxyl ligands is also demonstrated in the study by Lin *et al.*^42^, where ultrasonication of *h-*BN in water results in the formation of B-OH near the defect sites in BN. It is likely that these B-OH bonds are covalent in nature similar to the covalent hydroxylation of *h*-BN nanosheets reported by Sainsbury *et al.*^33^,^43^. The high temperature zones formed at the implosion sites during ultrasonication are expected to facilitate this chemical modification. Owing to the modified stoichiometry and presence of hydroxyl groups, we refer these nanosheets as chemically modified MgB_2_ nanosheets (CMMBs). It is expected that not all pristine MgB_2_ flakes will undergo this delamination; some flakes will undergo degradation to form insoluble products due to a chemical reaction with water^44^.

The hydroxyl groups are expected to ionize and impart a negative surface charge to these CMMBs similar to the negative surface charges acquired by graphene oxide sheets^45^,^46^. This is confirmed by measuring the zeta potential of the colloidal dispersion for a range of pH values (Fig. 3 top right inset). A maximum value of –32.6 is observed at pH 6.85. The negative surface charges are expected to impart moderate electrostatic stability to these nanosheets. The presence of hydroxyl groups also imparts hydrophilicity to the CMMBs. The CMMB dispersion was found to be stable for ~4 weeks after which visible white flocculates start appearing in the dispersion.

**Figure 4:**
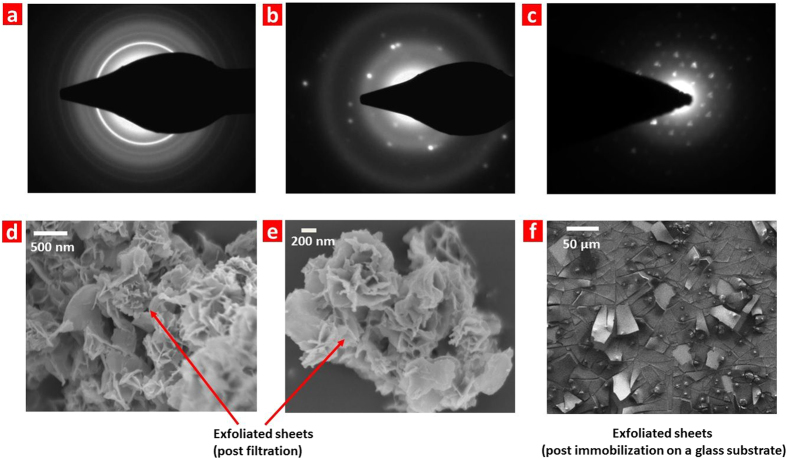
The SAED patterns and morphological characteristics of the CMMB nanosheets. Electron diffraction patterns taken from various positions of different nanosheets: **a**, most of the spectra suggest an amorphous nature of nanosheets **b**, **c**, some spectra indicate crystalline nature (incidence of such patterns is much lesser). **d,e**, FESEM images of nanosheets obtained by filtration of the aqueous dispersion. **f**, FESEM image of nanosheets immobilized by drop-casting the aqueous dispersion on a soda lime glass.

Crystallinity of the nanosheets was investigated by collecting selected area electron diffraction (SAED) patterns from various regions of several nanosheets. We observed three types of SAED patterns: (i) weak and diffused rings as shown in Fig. 4a, that indicates a loss of preferred stacking orientations, and hence an amorphous nature of these nanosheets. The disruption of long range ordering in crystal lattice is expected due to the functionalization and loss of Mg atoms from the layers. This is similar to the observation that graphite loses long range ordering upon exfoliation to graphene-oxide^47^,^48^.; (ii) random diffraction spots without any regular arrangement (Fig. 4b), which can be assigned to polycrystallinity; (iii) sharp six fold symmetry as observed in Fig. 4a which is inferred as a single crystalline nature^22^(the incidence of such a pattern was extremely less, see Supplementary Information, Fig. S6 for more representative SAED patterns). These observations suggest a non-uniform displacement of Mg atoms from the interspaces of boron honeycomb planes.

In order to determine the lateral sizes of nanosheets, FESEM images were procured from the powdered form of nanosheets (obtained by filtration of the aqueous dispersion) (Fig. 4d, e; Supplementary Information, Fig. S7). The filtered powder is found to consist of aggregated nanosheets, which appear like flowers, owing to the varying orientations in which nanosheets protrude from the aggregates. The nanosheets exhibited lateral sizes in the range of 1–50 μm. A layer of platinum coating was applied prior to FESEM imaging to reduce the charging effect that might render some nanosheets to appear thicker than expected^42^. We examined the pristine MgB_2_ powder under FESEM and found it to consist of thick flakes with large lateral sizes (<150 μm, shown in Supplementary Information, Fig. S8). Similarly, the sediments collected during centrifugation are found to consist of thicker flakes (See Supplementary Information, Fig. S8). This indicates that ultrasonication also induces a fragmentation of pristine MgB_2_ flakes similar to the sonication-induced fragmentation of graphite^49^,^50^. The nanosheets were immobilized by drop casting on a glass substrate which is found to result in an ordered arrangement as shown in Fig. 4f (see Supplementary information S10). The nanosheets are also accompanied by some globular nanostructures that are likely formed from the smaller sized crystals of MgB_2_ (shown in Supplementary Information, Fig. S9).

**Figure 5:**
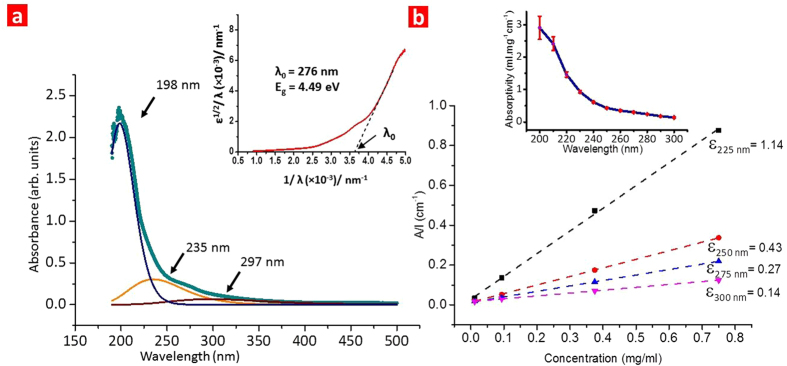
Optical properties of the colloidal dispersion containing CMMBs. **a**, Absorbance spectrum of the aqueous dispersion of MgB_2_ nanosheets (0.75 mg/ml, 1 cm optical path) at room temperature. The absorbance spectrum was deconvoluted mathematically (using Gaussian curve fitting) to three daughter spectra with individual peaks at 198 nm(blue), 235 nm(orange), and 297 nm(wine red). Inset shows the Tauc plot generated from the absorption spectrum of the CMMB nanosheet dispersion. The linear regime is extrapolated to obtain a gap wavelength (λ_g_) of 276 nm corresponding to which the optical band gap (E_g_) is 4.49 eV. **b**, Lambert-Beer plot for the aqueous dispersion of MgB_2_ nanosheets to estimate the absorptivity, ε(ml.mg^−1^cm^−1^) at different wavelengths(R^2^ > 0.99 in all cases). Inset shows the dependence of absorptivity on wavelength.

The colloidal dispersions were characterized by optical absorption spectroscopy as shown in Fig. 5a. The spectrum is found to be featureless in visible-IR region but exhibits a strong absorption pattern below 400 nm. The obtained absorption spectrum was deconvoluted to three daughter spectra using Gaussian distribution fit (method discussed in Supplementary Information, S12). The peaks of daughter spectra are located at ~198 nm, ~235 nm, and ~297 nm. It is expected that the introduction of hydroxyl groups will result in new electron states as compared to pristine MgB_2_. The boron honeycomb planes exhibit bonding orbitals (σ, π) and anti-bonding orbitals (σ*, π*). The introduction of hydroxyl groups will result in non-bonding (n) orbitals by virtue of the lone pair present on oxygen. The absorbance at ~198 nm is likely due to the π → π* electron transition. The broad shoulder peaks observed at ~235 nm and ~297 nm can be attributed to n → σ* and n → π* transitions^51^. Thus, the weak peaks are attributed to the oxygen functionalization on the nanosheets. An approximate optical band gap of the CMMB nanosheets was obtained by generating Tauc plot from the absorption spectrum (Fig. 5a inset). The linear regime in Tauc plot was fitted and extrapolated to obtain a gap wavelength (λ_g_) of ~276 nm that corresponds to an approximate optical band gap 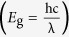 of 4.49 eV^52^.

The absorptivity of CMMB nanosheets was calculated by analysing a series of absorption spectra obtained for different concentrations of the nanosheet dispersion. Figure 5b shows absorbance per unit length (for four sample wavelengths) plotted against the concentration of dispersion. The slope of straight line fits is calculated (by using 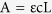) to obtain the values of absorptivity at each wavelength (Fig. 5b inset). The absorptivity is observed to decrease with an increase in wavelength. Its maximum value is recorded at λ = 200 nm as 2.9 ml mg^−1^ cm^−1^. This value is much lesser compared to the absorptivity values for other quasi 2D nanomaterials (graphene ε_660_ nm = 24.60 ml mg^−1^ cm^−1^; MoS_2_ ε_672_ nm = 34.00 ml mg^−1^ cm^−1^, WS_2_ ε_629_ nm = 27.50 ml mg^−1^ cm^−1^, *h-*BN ε_300_ nm = 23.67 ml mg^−1^ cm^−1^)^2^,^49^. This suggests that the CMMB nanosheets are several times more transparent than graphene and its reported analogs.

**Figure 6:**
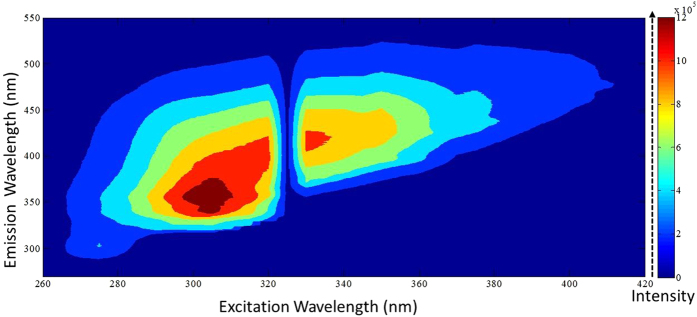
A map of photoluminescence intensity as a function of excitation and emission wavelengths for CMMB nanosheets. The emission intensity at various emission wavelengths (y-axis) is represented by the color scheme shown as a function of excitation wavelength (x-axis). A broad range of excitation wavelengths were used. The photoluminescence spectra is shown for excitation wavelengths 260 nm–420 nm.

The observation of new electron states in the nanosheets motivated us to investigate if this modified electron band structure can lead to a photoluminescence (PL) in the nanosheets. The nanosheet dispersions were irradiated with a broad range of excitation wavelengths and observed for any photoluminescence emissions. We observed stronger emissions for excitation wavelengths ranging from 260–420 nm and weaker emissions for excitation wavelengths between 420–500 nm. The emission intensity was found to be featureless for excitation wavelengths >500 nm. The wavelength of emission maxima was found to vary with the excitation wavelength suggesting an excitation-wavelength dependent PL (see Supplementary Information, Figs. S11 and S12 for representative plots). Figure 6 represents a map of PL intensity plotted as a function of excitation and emission wavelengths. The broad range of permissible transitions indicates that a number of new electron states have been generated in CMMBs as compared to pristine MgB_2_ that is known for exhibiting two band gaps of 2.2 meV and 7.1 meV^18^. Pristine MgB_2_ didn’t exhibit any native photoluminescence supporting that the PL is attained as a consequence of exfoliation (see Supplementary information S13 for more information).

The presence of a wide range of new electron states in CMMBs is attributed to (i) the different degrees of functionalization of nanosheets^53^, and (ii) crystal defects generated during extraction of Mg atoms^54^. The PL intensity is found to exhibit two maxima: the first maximum corresponds to λ_ex_ = 305 nm, λ_em_ = 358 nm; and the second maximum corresponds to λ_ex_ = 325 nm, λ_em_ = 411 nm. The emission at ~411 nm can be attributed to the presence of oxy-functional groups on the nanosheets. The functionality BO^-^ in hydroxylated *h-*BN nanosheet has been shown to result in similar PL with λ_em_ = 415 nm^55^. Further experimental investigations are required to obtain a deeper understanding of the nature of photoluminescence in CMMBs and the factors that govern it.

## Conclusion

We have demonstrated the feasibility of exfoliating MgB_2_ using the simple tool of ultrasonication in an aqueous phase. This ability to synthesize chemically modified MgB_2_ nanosheets will significantly add to the current state of knowledge on boron-based quasi 2-D nanostructures. It will be exciting to explore the electronic, mechanical and thermal properties of these Mg-deficient hydroxyl functionalized boron-based nanosheets. The possibility of tailoring the surface chemistry of these nanosheets makes these attractive candidates as fillers in polymer nano composites. The processable aqueous dispersions of these nanosheets can be utilized to form macrostructures that can leverage these nano interfaces. Further, an extremely small absorptivity combined with the photoluminescence makes these nanosheets promising constructs for optoelectronic applications. In addition, this study has evidenced that ultrasonication can result in exfoliating layered materials with inter layer binding forces stronger than the van der Waals forces. It will be promising to explore the possibility of exfoliating other metal borides, such as AlB_2_ and TiB_2_, which are isostructural to MgB_2_. We anticipate this study to potentially lead to the evolution of a new class of boron-based quasi 2-D nanomaterials.

## Methods

### Exfoliation of MgB_2_

Exfoliation was carried out by suspending 450 mg of MgB_2_ powder (–100 mesh size, Sigma Aldrich, purity ≥96%) in 150 ml water and exposing to ultrasonication using a 1 inch probe ultrasonicator (Sonic Vibracell-VC505, 500 watt, 20 kHz) for 30 minutes (amplitude: 30%, 10 sec on/off pulse). The resultant dark black suspension was allowed to stand for 24 hours. A distinct phase separation was observed as top phase (lighter in appearance) and bottom phase (darker in appearance). Without disturbing the sediments, decantation was carried out to separate the top phase. This top phase was exposed to two cycles of centrifugation at 1500 rpm for 45 minutes to pellet out any heavier particulate residues (Eppendorf centrifuge-5430R). After each centrifugation, top half of the dispersion was collected for further investigation.

### Lyophilization

The aqueous nanosheet dispersion (10 ml) was taken in a Falcon tube and frozen at –80 °C in Esco Lexicon ULT Freezer. The frozen sample was lyophilized (CHRIST, Alpha 2–4 LD plus lyophilizer) for ~48 hours until a white colored powder was observed.

### Dialysis

Dialysis was carried out by placing 40–50 ml of nanosheet dispersion inside a cellulosic dialysis membrane tube (Sigma Aldrich, 12 kDa). The opening of the tubes were tightly clipped (Snakeskin dialysis tubing clips) ensuring no leakage. The concentration gradient was maintained by keeping the dialysis tube inside a large beaker filled with deionized (DI) water. The DI water was changed every 3 hours and stirred magnetically to accelerate the process of dialysis. The dialysed sample was collected after 48 hours for further analysis.

### FESEM

FESEM was carried out on a JEOL (JSM-7600F) field emission scanning electron microscope, operated at 5–10 kV. Scanning electron microscopy (SEM)/energy dispersive X-ray (EDX) spectroscopy was used for measuring the elemental constituents by collecting the spectrum in silicon drift detection system. The powdered form of the nanosheet was sprinkled on the carbon tape while liquid sample was drop casted on the glass substrate before imaging.

### Optical Studies

The UV-vis absorption spectra were recorded on a spectrometer (Shimadzu, UV-1700 Pharma Spec UV-vis) over the range of 190–1100 nm in a quartz cuvette (1 cm). The different concentrations of the dispersion were prepared by serial dilutions of a stock dispersion prepared by re dispersing the lyophilized powder in DI water. Photoluminescence measurements were carried out with Fluorolog HORIBA Jobin Yvon, USA spectrofluorometer.

### TEM, HRTEM and SAED

Low-magnification transmission electron microscopy (TEM) images were acquired using a Philips Tecnai 20 TEM. High-resolution TEM (HRTEM) experiments were conducted on FEI Tecnai G2F*-*20 and JEOL, JEM-2100. The selected area electron diffraction (SAED) patterns were taken under both the TEMs. TEMs were operated at 200 kV in both cases. TEM/EDX analysis was conducted on a FEI Tecnai G2F*-*20 operated at 200 kV. TEM measurements were carried out by depositing the dispersion on to the surface of 300 mesh size 3 mm copper TEM grid supported with carbon film. The deposited sheets were allowed to dry under ambient conditions prior to examination.

### Attenuated Total Reflectance Fourier Transformed Infrared Spectroscopy (ATR-FTIR)

Infrared spectra were recorded in the region of 650–4000 cm^−1^ on a Thermo Scientific Nicolet iS10 FTIR spectrometer equipped with an ATR accessory. The lyophilized powder sample was prepared and kept inside vacuum before the analysis to avoid any contact with moisture. Multiple scanning was acquired to substantiate the result obtained.

### ICP-AES

The stoichiometric ratio of Mg and B in the nanosheets was obtained by measuring the concentration of Mg and B using ICP-AES (Optima 3300 DV, Perkin Elmer, US). Ten ml of the colloidal dispersion of nanosheets were used for the analysis and DI water was used as control during all measurements.

### Raman Spectroscopy

Raman spectroscopy was performed with a 785 nm laser source using Reinshaw Raman microscope. Typically 5 mg of lyophilized nanosheet powder and pristine MgB_2_ powder were used without any solvent and the laser source was focused on to the samples through 20X objective lens. Each spectrum consists of 5 accumulations with a 10 sec exposure per scan. Noise of the spectra was corrected using FFT filter in the Origin software.

### AFM

Samples for AFM analysis were prepared by spin coating (3000 rpm, 30 sec) 100 μL of the nanosheet dispersion on a freshly cleaved mica substrate and allowing it to dry in a desiccator for ~48 hours. The images were collected using Atomic force microscope from NT-MDT, Moscow, Russia. The AFM images were acquired in tapping mode with the aid of a silicon cantilever (spring constant 3.08 N/m and resonating frequency 140 kHz). The imaging was carried out at ambient room temperature and pressure, and all the images were processed using Nova 1.1.0.1780 software.

## Additional Information

**How to cite this article**: Das, S. K. *et al.* Aqueous dispersions of few-layer-thick chemically modified magnesium diboride nanosheets by ultrasonication assisted exfoliation. *Sci. Rep.* **5**, 10522; doi: 10.1038/srep10522 (2015).

## Supplementary Material

Supplementary Information

## Figures and Tables

**Table 1:**
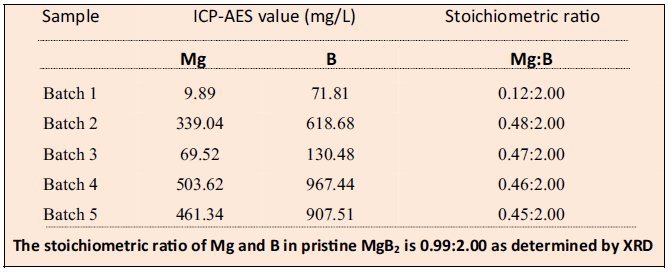
Stoichiometric ratio of Mg and B in the nanosheets obtained using ICP-AES analysis.

## References

[b1] Chhowalla M. *et al.* The chemistry of two-dimensional layered transition metal dichalcogenide nanosheets. Nat. Chem. 5, 263–275 (2013). 23511414 10.1038/nchem.1589

[b2] Coleman J. N. *et al.* Two-dimensional nanosheets produced by liquid exfoliation of layered materials. Science 331, 568–571 (2011). 21292974 10.1126/science.1194975

[b3] Nicolosi V., Chhowalla M., Kanatzidis M. G., Strano M. S. & Coleman J. N. Liquid exfoliation of layered materials. Science 340, 10.1126/science.1226419 (2013).

[b4] Naguib M. *et al.* Two-dimensional Transition Metal Carbides. ACS Nano 6, 1322–1331 (2012). 22279971 10.1021/nn204153h

[b5] Geim A. K. & Novoselov K. S. The rise of graphene. Nat. Mater. 6, 183–191 (2007). 17330084 10.1038/nmat1849

[b6] Wang Q. H., Kalantar-Zadeh K., Kis A., Coleman J. N. & Strano M. S. Electronics and optoelectronics of two-dimensional transition metal dichalcogenides. Nat. Nanotechnol. 7, 699–712 (2012). 23132225 10.1038/nnano.2012.193

[b7] Dean C. R. *et al.* Boron nitride substrates for high-quality graphene electronics. Nat. Nanotechnol. 5, 722–726 (2010). 20729834 10.1038/nnano.2010.172

[b8] Martin J. E. in Physics for Radiation Protection *Ch. 14* (Wiley-VCH Verlag GmbH, Weinheim, 2008).

[b9] Delaplane R. G., Dahlborg U., Howells W. S. & Lundström T. A neutron diffraction study of amorphous boron using a pulsed source. J. Non-Cryst. Solids 106, 66-69, 90229–3 (1988).

[b10] Wentorf R. H. Jr. Boron: Another Form. Science 147, 49–50 (1965). 17799779 10.1126/science.147.3653.49

[b11] Boustani I. in Chemical Modelling: Applications and Theory, Vol. 8 1–44 (The Royal Society of Chemistry, 2011).

[b12] Golberg D. *et al.* Boron nitride nanotubes and nanosheets. ACS Nano 4, 2979–2993 (2010). 20462272 10.1021/nn1006495

[b13] Zhi C., Bando Y., Tang C., Kuwahara H. & Golberg D. Large-scale fabrication of boron nitride nanosheets and their utilization in polymeric composites with improved thermal and mechanical properties. Adv. Mater. 21, 2889–2893 (2009).

[b14] Song L. *et al.* Large scale growth and characterization of atomic hexagonal boron nitride layers. Nano Lett. 10, 3209–3215 (2010). 20698639 10.1021/nl1022139

[b15] Nag A. *et al.* Graphene analogues of BN: novel synthesis and properties. ACS Nano 4, 1539–1544 (2010). 20128601 10.1021/nn9018762

[b16] Gao R. *et al.* High-yield synthesis of boron nitride nanosheets with strong ultraviolet cathodoluminescence emission. J. Phys. Chem. C 113, 15160–15165 (2009).

[b17] Piazza Z. A. *et al.* Planar hexagonal B36 as a potential basis for extended single-atom layer boron sheets. Nat. Commun. 5, 10.1038/ncomms4113 (2014). 24445427

[b18] Pissas M. in Low-Dimensional Solids 229–286 (John Wiley & Sons, Ltd, 2010).

[b19] Medvedeva N. I., Ivanovskii A. L., Medvedeva J. E. & Freeman A. J. Electronic structure of MgB_2_ and related binary and ternary borides. Phys. Rev. B 64, 020502 (2001).

[b20] Ivanovskii A. L. M., I. Interatomic interactions and electronic structure of hexagonal magnesium, aluminum and silicon diborides: Ab initio full-potential LMTO calculations. Russ. J. Inorg. Chem. 45, 1355–1361 (2000).

[b21] Kortus J., Mazin I. I., Belashchenko K. D., Antropov V. P. & Boyer L. L. Superconductivity of metallic boron in MgB_2_. Phys. Rev. Lett. 86, 4656–4659 (2001). 11384307 10.1103/PhysRevLett.86.4656

[b22] Chen W., Liu W., Chen C., Wang R. & Feng Q. Single-crystal MgB_2_ hexagonal microprisms viahybrid physical-chemical vapor deposition. Cryst eng comm 13, 3959–3961 (2011).

[b23] Carenco S., Portehault D., Boissière C., Mézailles N. & Sanchez C. Nanoscaled metal borides and phosphides: recent developments and perspectives. Chem. Rev. 113, 7981–8065 (2013). 23767879 10.1021/cr400020d

[b24] Greim J. & Schwetz K. A. in Ullmann’s Encyclopedia of Industrial Chemistry (Wiley-VCH Verlag GmbH & Co. KGaA, 2000).

[b25] Sen S., Ozbek I., Sen U. & Bindal C. Mechanical behavior of borides formed on borided cold work tool steel. Surf. Coat. Tech. 135, 173–177 (2001).

[b26] Mori T. & Nishimura T. Thermoelectric properties of homologous p- and n-type boron-rich borides. J. Solid State Chem. 179, 2908–2915 (2006).

[b27] Turchanin A. *et al.* One nanometer thin carbon nanosheets with tunable conductivity and stiffness. Adv. Mater. 21, 1233–1237 (2009).

[b28] Cheng C. & Li D. Solvated graphenes: an emerging class of functional soft materials. Adv. Mater. 25, 13–30 (2013). 23180382 10.1002/adma.201203567

[b29] Wang Z. *et al.* Facile, mild and fast thermal-decomposition reduction of graphene oxide in air and its application in high-performance lithium batteries. Chem. Commun. 48, 976–978, (2012). 10.1039/c2cc16239c 22159368

[b30] Ramanathan T. *et al.* Functionalized graphene sheets for polymer nanocomposites. Nat. Nanotechnol. 3, 327–331 (2008). 18654541 10.1038/nnano.2008.96

[b31] Schniepp H. C. *et al.* Functionalized Single Graphene Sheets Derived from Splitting Graphite Oxide. J. Phys. Chem. B Lett. 110, 8535–8539 (2006). 10.1021/jp060936f 16640401

[b32] Nyquist R. A. & Kagel R. O. in Handbook of Infrared and Raman Spectra of Inorganic Compounds and Organic Salts (eds Nyquist Richard A. & Ronald O. Kagel) 1–18 (Academic Press, 1971).

[b33] Sainsbury T. *et al.* Oxygen radical functionalization of boron nitride nanosheets. J. Am. Chem. Soc. 134, 18758–18771 (2012). 23101481 10.1021/ja3080665

[b34] Rodríguez M. G., Kharissova O. V. & Ortiz-Mendez U. Formation of boron carbide nanofibers and nanobelts from heated by microwave. Rev. Adv. Mater. Sci 7, 55 (2004).

[b35] Peak D., Luther G. W. III & Sparks D. L. ATR-FTIR spectroscopic studies of boric acid adsorption on hydrous ferric oxide. Geochim. Cosmochim. Ac. 67, 2551–2560 (2003).

[b36] Bellamy L. J., Gerrard W., Lappert M. F. & Williams R. L. Infrared spectra of boron compounds. J. Chem. Soc. 2412–2415 (1958).

[b37] Tsou H. T. & Kowbel W. Design of multilayer plasma-assisted CVD coatings for the oxidation protection of composite materials. Surf. Coat. Tech. 79, 139–150 (1996).

[b38] Alarco J. A., Chou A., Talbot P. C. & Mackinnon I. D. R. Phonon modes of MgB2: super-lattice structures and spectral response. Phys. Chem. Chem. Phys. 16, 24443–24456 (2014). 25308214 10.1039/c4cp03449j

[b39] Hlinka J. *et al.* Phonons in MgB_2_ by polarized Raman scattering on single crystals. Phys. Rev. B 64, 140503 (2001).

[b40] Yi M. *et al.* Hydrodynamics-assisted scalable production of boron nitride nanosheets and their application in improving oxygen-atom erosion resistance of polymeric composites. Nanoscale 5, 10660–10667 (2013). 24057073 10.1039/c3nr03714b

[b41] Naguib M. *et al.* Two-dimensional nanocrystals produced by exfoliation of Ti_3_AlC_2_. Adv. Mater. 23, 4248–4253 (2011). 21861270 10.1002/adma.201102306

[b42] Lin Y. *et al.* Aqueous dispersions of few-layered and monolayered hexagonal boron nitride nanosheets from sonication-assisted hydrolysis: critical role of water. J. Phys. Chem. C 115, 2679–2685 (2011).

[b43] Silberberg M. S. & Weberg E. B. Student Study Guide to accompany Chemistry: The Molecular Nature of Matter and Change. (McGraw-Hill Higher Education, 2009).

[b44] Zhai H. *et al.* Degradation of superconducting properties in MgB_2_ films by exposure to water. Supercond. Sci. Tech. 14, 425 (2001).

[b45] Li D., Muller M. B., Gilje S., Kaner R. B. & Wallace G. G. Processable aqueous dispersions of graphene nanosheets. Nat. Nanotechnol. 3, 101–105 (2008). 18654470 10.1038/nnano.2007.451

[b46] Zhao G. *et al.* Preconcentration of U(vi) ions on few-layered graphene oxide nanosheets from aqueous solutions. Dalton T. 41, 6182–6188 (2012). 10.1039/c2dt00054g 22473651

[b47] Williams D. & Carter C. B. in Transmission Electron Microscopy Ch. 1, 3–22 (Springer US, 2009).

[b48] McAllister M. J. *et al.* Single sheet functionalized graphene by oxidation and thermal expansion of graphite. Chem. Mater. 19, 4396–4404 (2007).

[b49] Hernandez Y. *et al.* High-yield production of graphene by liquid-phase exfoliation of graphite. Nat. Nanotechnol. 3, 563–568 (2008). 18772919 10.1038/nnano.2008.215

[b50] Khan U., O’Neill A., Lotya M., De S. & Coleman J. N. High-concentration solvent exfoliation of graphene. Small 6, 864–871 (2010). 20209652 10.1002/smll.200902066

[b51] Clark J. UV-Visible absorption spectra. (2007). Available at: http://www.chemguide.co.uk/analysis/uvvisible/theory.html (Accessed: 4th September 2014)

[b52] Ci L. *et al.* Atomic layers of hybridized boron nitride and graphene domains. Nat. Mater. 9, 430–435 (2010). 20190771 10.1038/nmat2711

[b53] Luo Z., Vora P. M., Mele E. J., Johnson A. T. C. & Kikkawa J. M. Photoluminescence and band gap modulation in graphene oxide. Appl. Phys. Lett. 94, 111909 (2009).

[b54] Tongay S. *et al.* Defects activated photoluminescence in two-dimensional semiconductors: interplay between bound, charged, and free excitons. Sci. Rep. 3, 10.1038/srep02657 (2013). PMC3772378 24029823

[b55] Tang C., Bando Y., Zhi C. & Golberg D. Boron-oxygen luminescence centres in boron-nitrogen systems. Chem. Commun. 4599–4601, 10.1039/B711807D (2007). 17989804

