# Supplementary material for: Aqueous dispersions of few-layer-thick chemically modified magnesium diboride nanosheets by ultrasonication assisted exfoliation
Source: Sci Rep. 2015 Jun 4;5:10522. doi: 10.1038/srep10522 (PMC4603704; doi:10.1038/srep10522)
Supplement: Supplementary Information [file srep10522-s1.pdf]

## Supplementary Information for
# Aqueous dispersions of few-layer-thick chemically modified magnesium diboride nanosheets by ultrasonication assisted exfoliation

Saroj Kumar Das[1], Amita Bedar[1], Aadithya Kannan[2] and Kabeer Jasuja[1]*

[1]Discipline of Chemical Engineering, Indian Institute of Technology Gandhinagar, Ahmedabad, Gujarat 382424, India

[2] Department of Chemical Engineering, Indian Institute of Technology Kharagpur, Kharagpur, 721302, India

* kabeer@iitgn.ac.in

### S1 TEM images of exfoliated $MgB_2$ nanostructures.

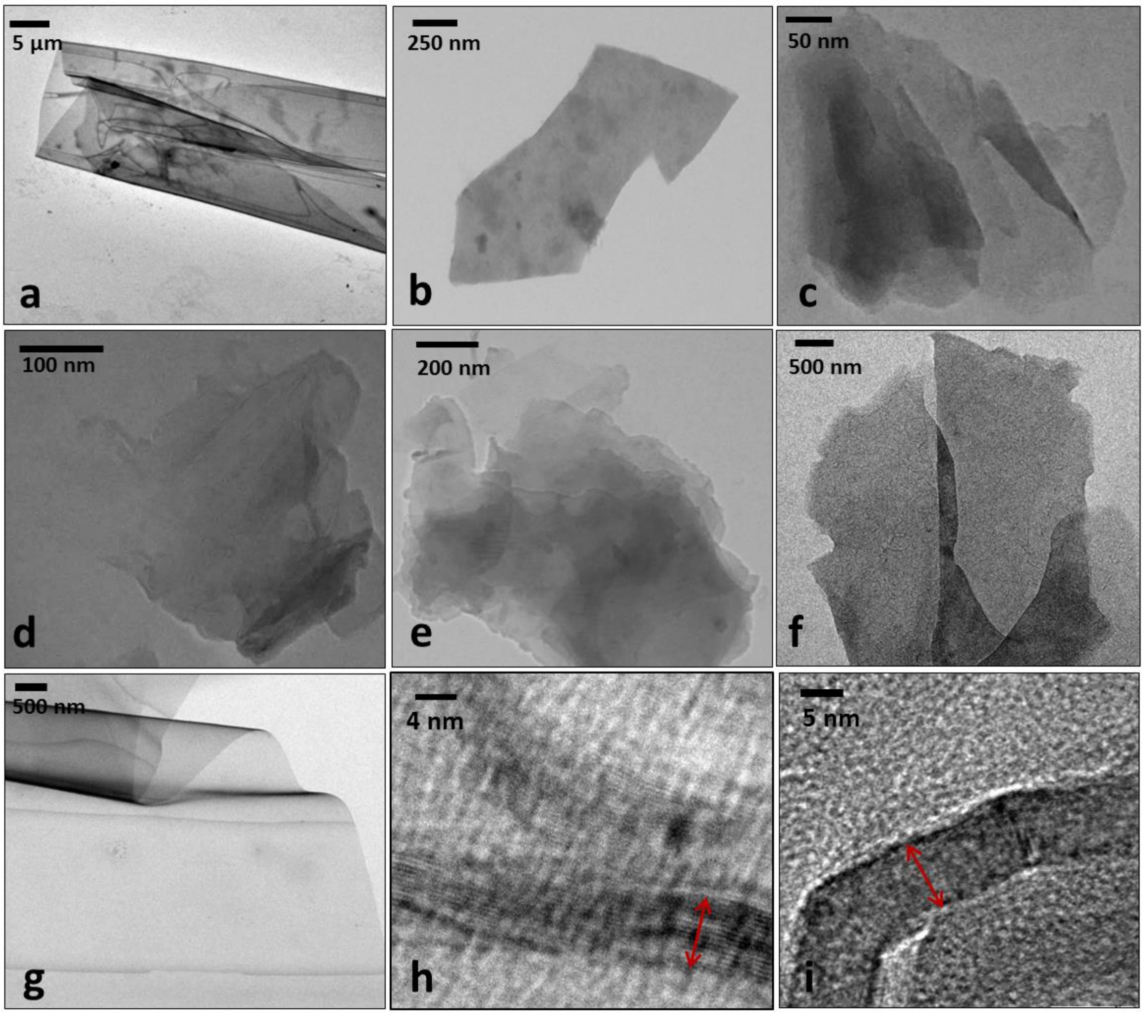


**Figure S1** TEM and HRTEM images of nanosheets immobilized from the colloidal dispersion

We performed TEM analysis of the dispersed phase by drop casting the aqueous dispersion on TEM grids. Most of the nanostructures are found to exhibit a quasi-planar morphology with transverse section representing the few layered structure shown in Figure S1 h and i. We refer these

nanostructures as nanosheets. Some nanosheets are found to exhibit folds near their edges (Figure S1a), while some nanosheets are found to overlap each other (Figure S1c-g). The lateral sizes of the nanosheets are found to be >1µm.

## S2 TEM and AFM images

By analysing a large number of TEM images we found several nanosheets to exhibit a crumpled morphology (Figure S2 a-d). Some of these nanosheets are extremely crumpled, whereas some are moderately crumpled. The darker regions observed on the surfaces of some nanosheets likely indicate extremely crumpled up regions. AFM was used to measure the thickness of nanosheets. Figure S2g-h represents the selected area scanning of the nanosheet shown in Figure 2g-h (main manuscript file). The lateral dimension of these nanosheets is found to be in the range of few nanometres to few micrometres. Height profiles are collected from different region of the nanosheet ans shown in in insets i, ii, and iii suggest the thickness of individual nanosheet in few nanometres. It can be clearly seen that a sudden fall in step height has occurred during the measurement. This is likely due to the presence of nanosheets overlapping on each other.

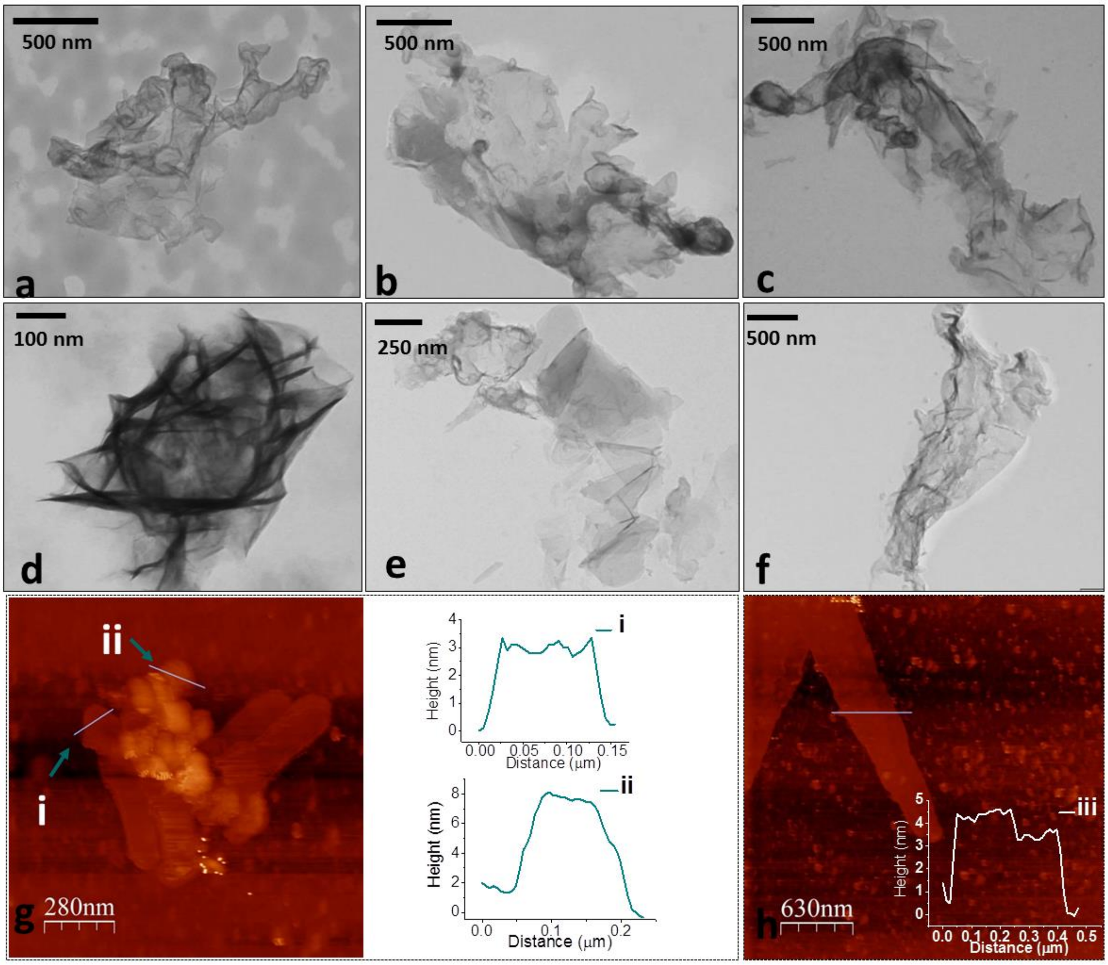


**Figure S2** TEM images of nanosheets that exhibit crumpling: a-d, Extremely crumpled nanosheets. e and f, Moderately crumpled nanosheets, g&h, AFM images of the nanosheets with corresponding height profiles (insets i,ii and iii) indicating the thickness in nanometers.

## S3 EDX analysis of nanosheets obtained under HRTEM

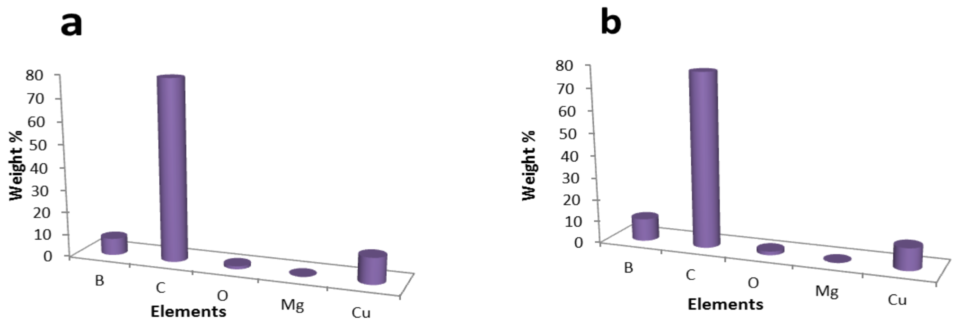


**Figure S3** Relative fraction of elements in nanosheets observed by an analysis of Energy Dispersive X-ray(EDX) spectra obtained from different regions of nanosheets.

Figure S3a and b represent the relative fraction of elements present in nanosheets as obtained from an EDX analysis under HRTEM. The spectra indicate moderate signal of boron (B) and relatively weak signal of magnesium (Mg) and oxygen (O). The weak signal of Mg suggests that Mg atoms are being displaced from the parent crystal structure during the exfoliation. The carbon (C) and copper (Cu) signals are attributed to the TEM copper grid carrying the carbon support film.

## S4 Determining the stoichiometric ratio of Mg and B in the nanosheets using Inductively Coupled Plasma-Atomic Electron Spectroscopy(ICP-AES)

The stoichiometric ratios of Mg and B in the dispersed nanosheets were obtained by ICP-AES analysis of the colloidal dispersion. The concentrations of Mg and B (mg/L) were obtained by ICP-AES analysis of three different batches of dispersion. Before the ICP-AES analysis, the dispersions were dialyzed using cellulosic membrane (12 kDa) to ensure that the signals are arising from the nanosheets and not any residual ions.

The stoichiometric ratio is calculated from ICP-AES values by using the following equation:

$$\frac{\textit{Concentration of Mg obtained by ICP-AES}}{\textit{Atomic weight of Mg}} : \frac{\textit{Concentration of B obtained by ICP-AES}}{\textit{Atomic weight of B}} \quad (1)$$

For example, the stoichiometric ratio for Batch 1 can be calculated as: $\frac{9.89}{24.30} : \frac{71.81}{10.81} = 0.12 : 2.00$

Similarly, the stoichiometric ratio for Batch 2 and 3 was calculated and listed in Table 1 (as shown in the main file).

## S5 EDX (FESEM) and Raman spectroscopy studies of CMMB nanosheets and pristine $MgB_2$

The water dispersed nanosheets were immobilized on a glass substrate by drop casting for FESEM imaging and EDX analysis. The relative weight percentage of the elements is shown in Figure S4. The spectrum indicates strong signal of oxygen, moderate signal of boron, and relatively weak signals of Mg and Si. The strong signal of oxygen and presence of Si is attributed to the underlying glass substrate ($SiO_2$). The nanosheets are also expected to contribute to the oxygen signal. The EDX spectrum of pristine $MgB_2$ was obtained by depositing the source $MgB_2$ powder on a conductive carbon tape. The relative concentration of Mg is found to be significantly higher as compared to the concentration of Mg in exfoliated nanosheet. The relatively lesser weight percentage of Mg in CMMBs corroborates the finding that Mg atoms are being displaced during ultrasonication.

The weak signal of oxygen in the source $MgB_2$ powder is likely originating from the MgO contamination as reported earlier by Singh et al. and Rajput et al[1,2].

Figure S4c compares the Raman spectra of pristine $MgB_2$ and CMMB nanosheets after excitation with 785 laser sources. The Raman spectrum for pristine $MgB_2$ shows multiple peaks, which agrees reasonably well with the observations by Alarco *et al.* at the same excitation wavelength[3]. Furthermore, the Raman active $E_{2g}$ mode observed in the range of 550-650 $cm^{-1}$ corroborates the presence of B-B in plane stretching mode of pristine $MgB_2$ and CMMBs. This is in accordance with the several reported observations, which are typically in the range of ~570-630 $cm^{-1}$ [3-5]. It can be clearly seen from the Raman spectra of CMMBs that the broadening and shifting of peaks has occurred as compared to the peaks of pristine $MgB_2$. This is likely due to the two main reasons: (i) reduction of thickness (similar to the observation in $TiO_2$ nanosheets where $E_g$ vibrational mode broadens and shifts to higher wavenumber with decrease in the thickness of nanosheet)[6]; (ii) presence of functional groups on the nanosheet (as reported by Georgakilas *et al.* and Kudin *et al.*, where functionalization of graphene modifies the planar carbon atoms and is responsible for broadening and shifting of in plane vibrational G band/$E_{2g}$ phonon)[7,8]. We hope to carry out a detailed study in near future to identify the reasons for the shift and further understand the behaviour of the other peaks commonly encountered in the Raman spectra of $MgB_2$ as shown elsewhere[3].

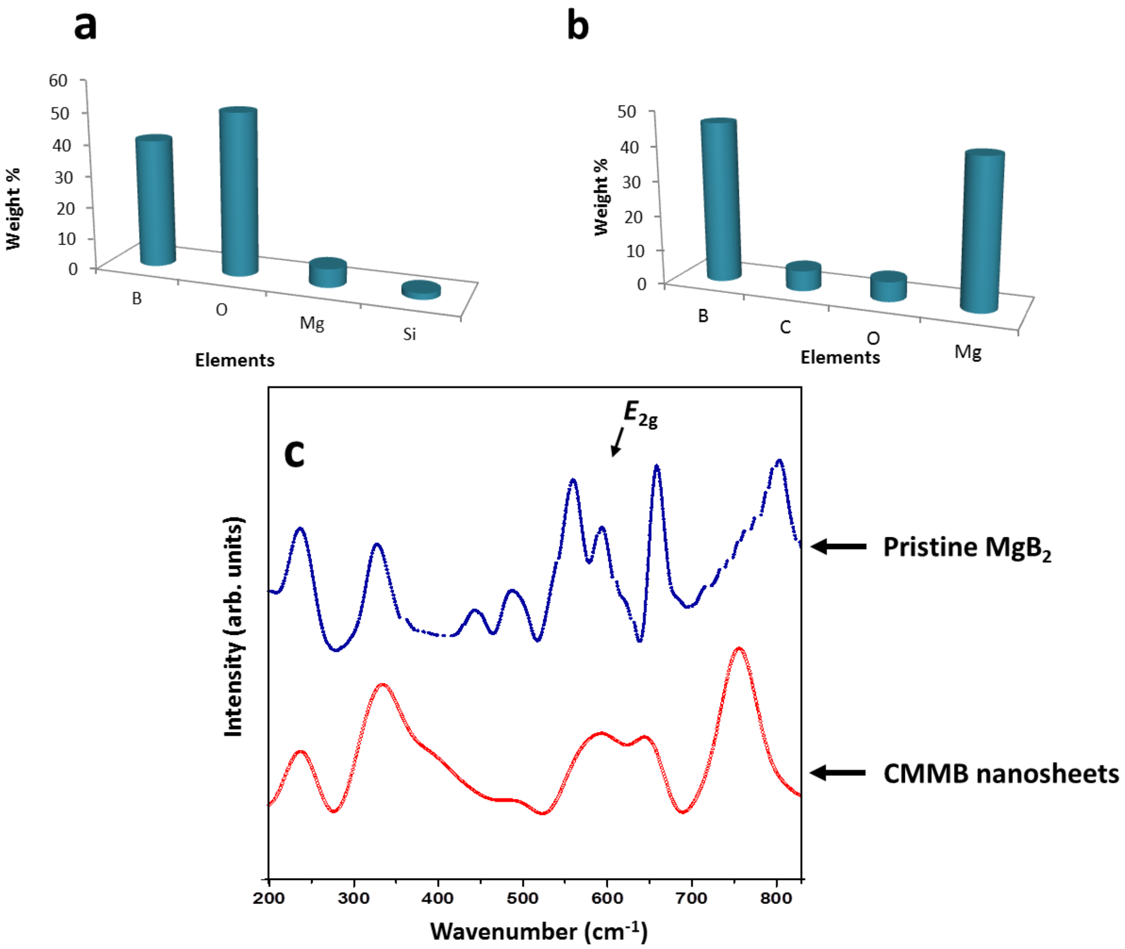


**Figure S4 a)**Relative fraction of elements in nanosheets observed in the EDX spectra obtained from nanosheets immobilized on a SEM substrate (glass), **b)** EDX spectrum of pristine $MgB_2$ deposited on a carbon tape, c) Raman spectra of pristine $MgB_2$(top) and CMMB nanosheets(bottom). The Raman frequency was calibrated using the 520 $cm^{-1}$ peak of the Si.

## S6 TEM images depicting delamination

We captured some TEM images of nanosheets that appeared to be in the process of being exfoliated from the parent structure (Figure S5). It is observed that extremely crumpled nanosheets are about to detach from a micron-sized parent flake.

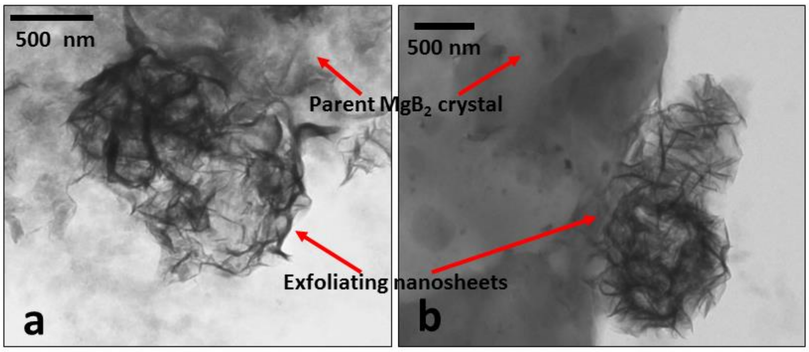


**Figure S5** TEM images of extremely crumpled nanosheets about to delaminate from its parent structure

## S7 SAED studies

The SAED patterns obtained during TEM analysis suggest that most of these nanosheets are amorphous in nature. Some nanosheets display a random arrangement of diffraction spots suggesting the presence of polycrystalline regions (figure S6e and f).

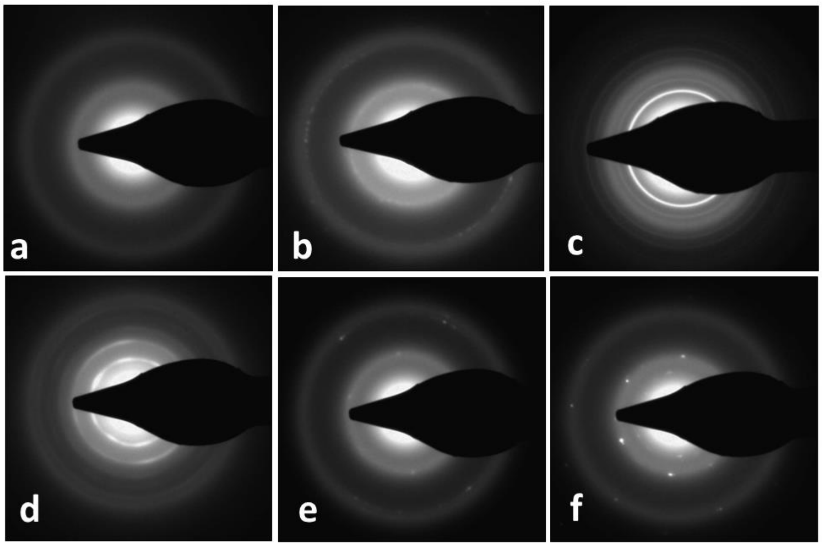


**Figure S6** SAED pattern obtained on various regions of several nanosheet. a-d, amorphous nature of the nanosheets. e&f, crystalline nature of nanosheets

## S8 FESEM images on filtered nanosheet powder.

CMMB dispersions were filtered through Whatman filter paper (catalogue no. 1001-125, pore size 11μm). The filtered solution was dried completely in ambient air before the FESEM imaging. A thin layer of platinum was coated prior to imaging to reduce the charging. The FESEM images indicate that the exfoliated nanosheets aggregate upon filtration (Figure S7).

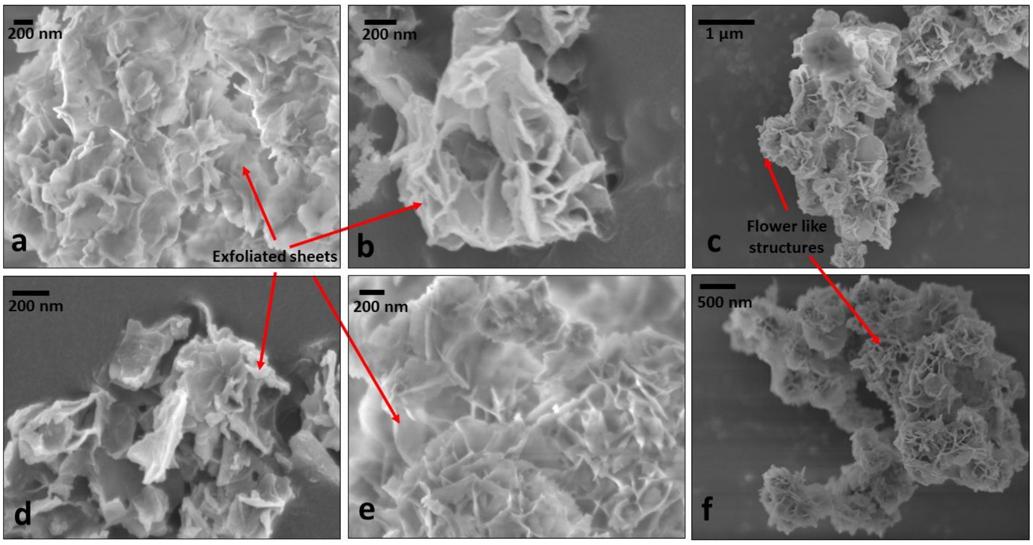


**Figure S7** FESEM images of powder obtained from filtration of the nanosheet dispersion

## S9 FESEM images of pristine Magnesium diboride powder and sediments collected after the sonication.

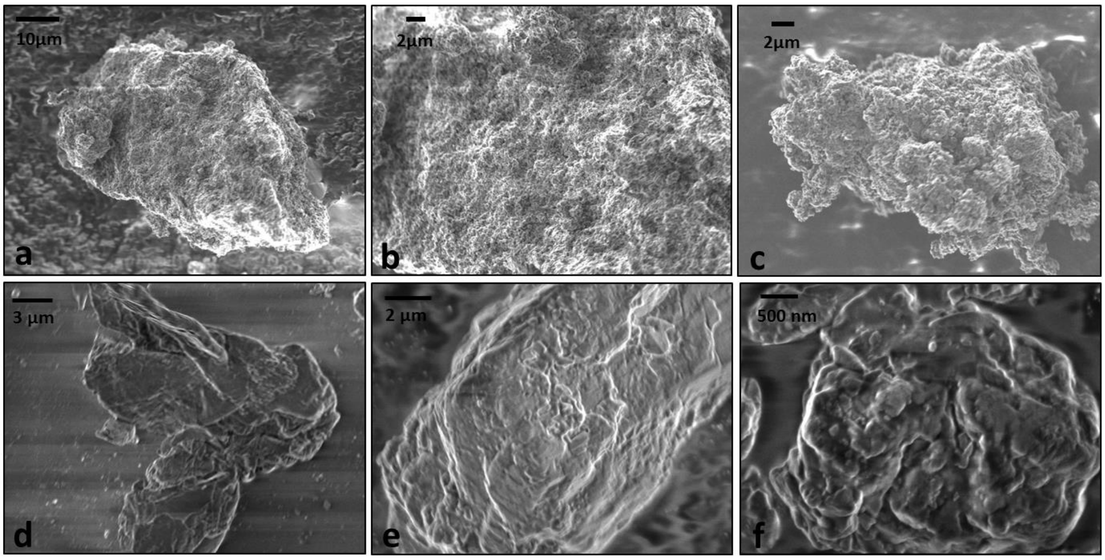


**Figure S8** FESEM images of pristine Magnesium diboride powder (a-c), and sediments after ultra sonication of Magnesium diboride (d-f).

The FESEM images of pristine $MgB_2$ powder shown in figure S8a-c suggest the micron scale thickness of the parent material. The sediments were collected by allowing the ultrasonicated aqueous $MgB_2$ dispersion to settle for 24 hours. The bottom part of the solution was air dried to collect the powdered form of the sediments for the analysis. The images from sediments confirm that most of these particles are in the range of micrometers.

## S10 Preparation of sample for immobilization on glass substrate.

The colloidal dispersion was prepared as discussed in Methods (synthesis of $MgB_2$ nanosheets). Glass substrate of 1cm x 1 cm was cleaned using a soap solution followed by acetone. The cleaned substrate was allowed to dry by spray drying. Colloidal dispersion of 100 µl was drop-

casted on to the glass substrate. Without disturbing, the sample was kept inside desiccator under vacuum environment for ensuring complete drying overnight before imaging.

## S11 FESEM images showing globular nanostructures.

The lyophilized forms of chemically modified nanosheets are observed under FESEM as shown in figure S9. Some globular microstructures are observed in addition to the nanosheets.

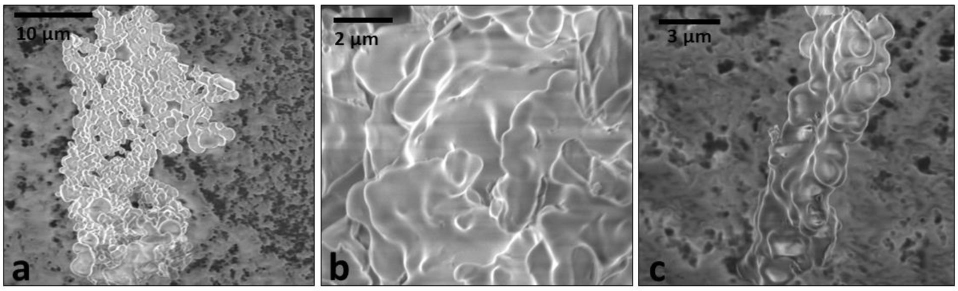


**Figure S9** FESEM images of the lyophilized dispersion depicting globular microstructures

## S12 Deconvolution using Gaussian distribution

The deconvolution of UV-visible absorption spectrum into individual bands was carried out by the help of Excel (Microsoft-2010) using the Solver tool[9]. This tool was used to fit the spectroscopy data to a sum of Gaussian functions using the following equation:

$$A = \sum_{i=1}^{n} A_i \, exp\left[-\frac{1}{2}\left\{\frac{1}{s_i}\left(\frac{1}{\lambda_{exp}} - \frac{1}{\lambda_i}\right)\right\}^2\right] \quad (2)$$

($A_i$ indicates the value of absorbance maximum, $s_i$ indicates value of bandwidth (width at half maxima), $\lambda_i$ indicate wavelength corresponding to $A_i$, and n indicates the number of constituent spectra that appear to form the original spectrum).The original UV-vis spectrum of nanosheets appears to be constituted from three absorption spectra suggesting n=3. Three sets of initial values for $A_i$, $s_i$, and $\lambda_i$ are considered as a first guess for the individual bands. Figure S10a presents an example of a first guess for the deconvolution process. Using Solver, these values are then iterated to best-fit the original UV-vis spectrum as sum of three Gaussian functions. Subsequent error is minimized with the help of Solver from approximated initial values. The final resolved spectrum is deduced as three distinct bands of blue, orange and wine red shown in figure S10b.

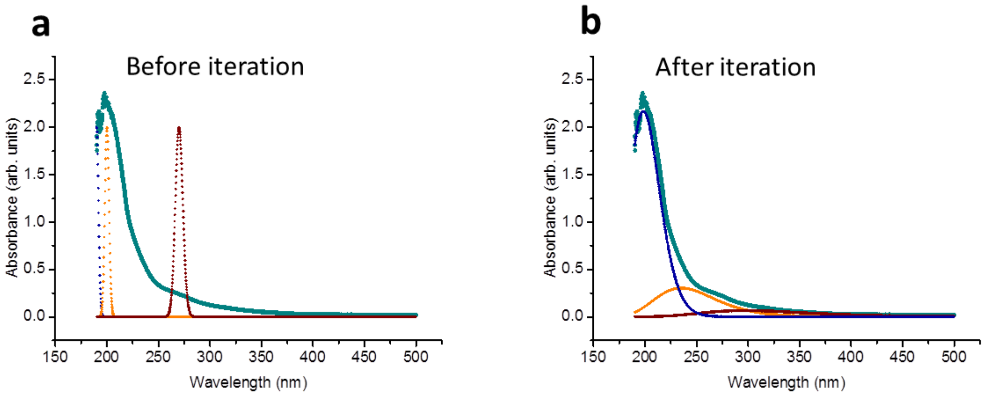


**Figure S10** Plot representing deconvolution a) before iteration, b) after iteration

## S13. Photoluminescence measurements

In a typical measurement, 4 ml of nanosheet dispersion was placed in a quartz cuvette and irradiated with a Xenon excitation source. The wavelength of the excitation light ranged from 190-500 nm. Strong emissions were observed for excitation wavelengths ranging from 260-420 nm, whereas weak emissions were obtained for excitation wavelengths between 420-500 nm. The emission intensity was found to be featureless for excitation wavelengths >500 nm. The corresponding emission spectra were recorded for each excitation wavelength. Example emission intensity plots are shown for three different excitation wavelengths of a) 305 nm, b) 325 nm and c) 390 nm on Figure S11. Emission intensity for all the excitation wavelengths are plotted to obtain a 3-D map of photoluminescence intensity as a function of excitation and emission wavelengths as shown in figure S12. The 2D version of the figure S12 is shown as Figure 6 in the main file. In order to test the photoluminescence property of pristine $MgB_2$, ~5 mg of powder sample was excited with the same range (190-500 nm) of excitation wavelengths. The emission intensity was found to be featureless for the entire set of excitation wavelengths. This is represented by showing the blank emission spectra for pristine $MgB_2$ at three sample excitation wavelengths of d) 305 nm, e) 325 nm and f) 390 nm in Figure S11.

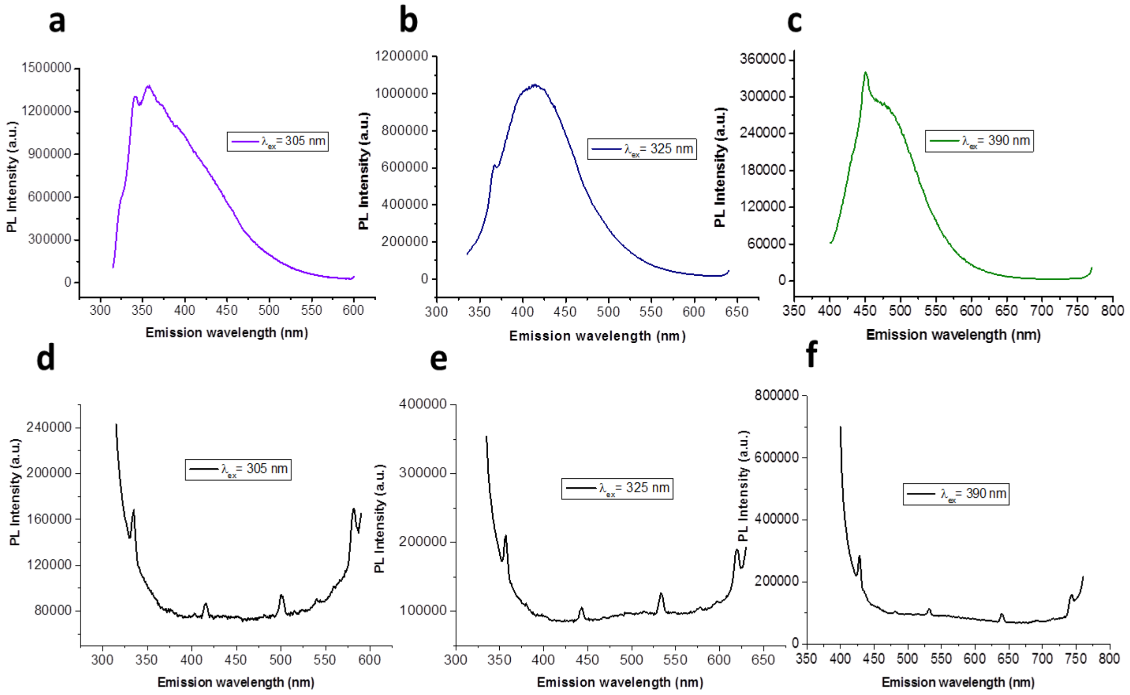


**Figure S11** Photoluminescence emission spectra of CMMBs obtained for different excitation wavelength a) 305 nm, b) 325 nm, and c) 390 nm. Photoluminescence emission spectra of pristine $MgB_2$ obtained at three excitation wavelengths d) 305 nm, e) 325 nm, and f) 390 nm.

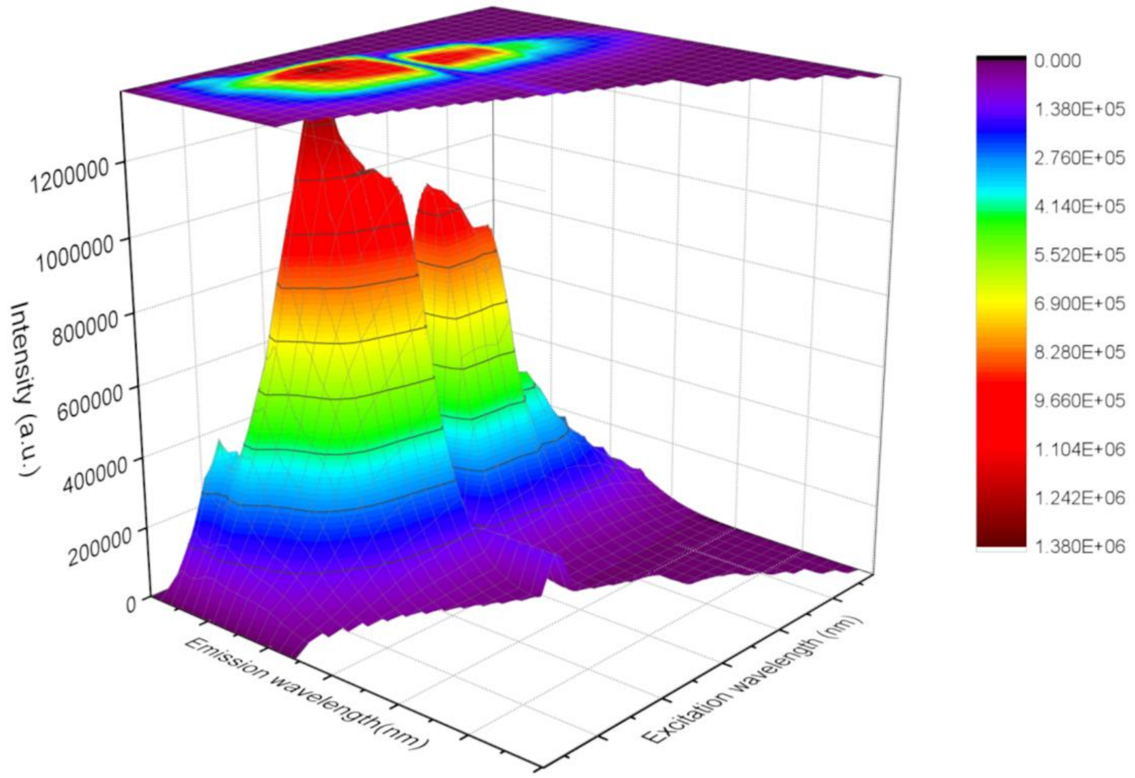


**Figure S12** Map of photoluminescence intensity (3D) as a function of excitation and emission wavelengths for CMMB nanosheets.

## S14. XRD plot of pristine $MgB_2$

The X-ray diffraction analysis of pristine $MgB_2$ powder was obtained in diffractometer (Bruker AXS, Germany). The plot confirms the crystalline nature of $MgB_2$ powder as shown in figure S13[10]. The stoichiometric ratio of Magnesium and Boron in pristine $MgB_2$ was predicted as 0.99:2.00 by comparing the spectrum against data from the international centre for diffraction database.

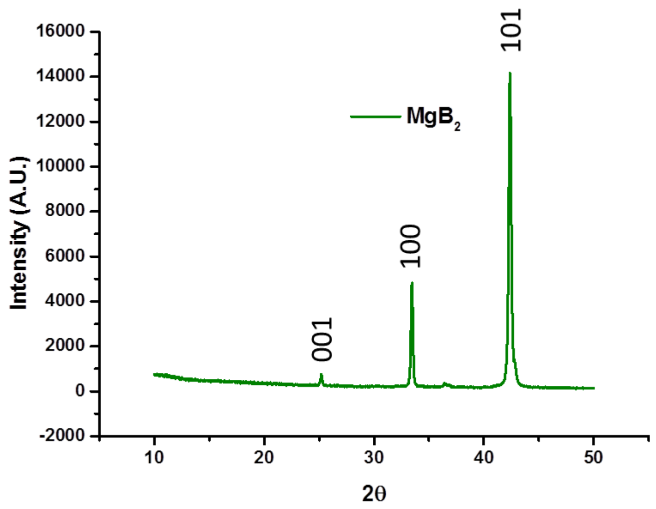


**Figure S13** XRD analysis of pristine $MgB_2$

References

1 Singh, D. K., Tiwari, B., Jha, R., Kishan, H. & Awana, V. P. S. Role of MgO impurity on the superconducting properties of $MgB_2$. *Physica C: Supercond.* **505**, 104-108 (2014).

2 Rajput, S., Chaudhary, S., Kashyap, S. C. & Srivastava, P. On the study of phase formation and critical current density in superconducting $MgB_2$. *Bull. Mater. Sci.* **29**, 207-211 (2006).

3 Alarco, J. A., Chou, A., Talbot, P. C. & Mackinnon, I. D. R. Phonon modes of MgB2: super-lattice structures and spectral response. *Phys. Chem. Chem. Phys.***16**, 24443-24456 (2014).

4 Hlinka, J. *et al.* Phonons in $MgB_2$by polarized Raman scattering on single crystals. *Physical Review B* **64**, 140503 (2001).

5 Bohnen, K. P., Heid, R. & Renker, B. Phonons in $MgB_2$ by polarized Raman scattering on single crystals. *Phys. Rev. Lett.* **86**, 5771-5774 (2001).

6 Yang, X. H. *et al.* Ultra-thin anatase $TiO_2$ nanosheets dominated with {001} facets: thickness-controlled synthesis, growth mechanism and water-splitting properties. *Cryst eng comm* **13**, 1378-1383 (2011).

7 Haubner, K. *et al.* The Route to Functional Graphene Oxide. *Chem Phys Chem* **11**, 2131-2139 (2010).

8 Georgakilas, V. *et al.* Functionalization of Graphene: Covalent and Non-Covalent Approaches, Derivatives and Applications. *Chem. Rev.* **112**, 6156-6214, (2012).

9 Billo, E. J. *Excel for chemists: a comprehensive guide*. (John Wiley & Sons, 2004).

10 Kováč, P. *et al.* The role of MgO content in ex situ MgB2 wires. *Supercond. Sci. Tech.* **17**, L41 (2004).
